# *SlDEAD31*, a Putative DEAD-Box RNA Helicase Gene, Regulates Salt and Drought Tolerance and Stress-Related Genes in Tomato

**DOI:** 10.1371/journal.pone.0133849

**Published:** 2015-08-04

**Authors:** Mingku Zhu, Guoping Chen, Tingting Dong, Lingling Wang, Jianling Zhang, Zhiping Zhao, Zongli Hu

**Affiliations:** Key Laboratory of Biorheological Science and Technology (Chongqing University), Ministry of Education, Bioengineering College, Chongqing University, Chongqing, 400044, People’s Republic of China; University of Delhi South Campus, INDIA

## Abstract

The DEAD-box RNA helicases are involved in almost every aspect of RNA metabolism, associated with diverse cellular functions including plant growth and development, and their importance in response to biotic and abiotic stresses is only beginning to emerge. However, none of DEAD-box genes was well characterized in tomato so far. In this study, we reported on the identification and characterization of two putative DEAD-box RNA helicase genes, *SlDEAD30* and *SlDEAD31* from tomato, which were classified into stress-related DEAD-box proteins by phylogenetic analysis. Expression analysis indicated that *SlDEAD30* was highly expressed in roots and mature leaves, while *SlDEAD31* was constantly expressed in various tissues. Furthermore, the expression of both genes was induced mainly in roots under NaCl stress, and *SlDEAD31* mRNA was also increased by heat, cold, and dehydration. In stress assays, transgenic tomato plants overexpressing *SlDEAD31* exhibited dramatically enhanced salt tolerance and slightly improved drought resistance, which were simultaneously demonstrated by significantly enhanced expression of multiple biotic and abiotic stress-related genes, higher survival rate, relative water content (RWC) and chlorophyll content, and lower water loss rate and malondialdehyde (MDA) production compared to wild-type plants. Collectively, these results provide a preliminary characterization of *SlDEAD30* and *SlDEAD31* genes in tomato, and suggest that stress-responsive *SlDEAD31* is essential for salt and drought tolerance and stress-related gene regulation in plants.

## Introduction

Plants, as sessile organisms, are continuously exposed to various environmental stresses such as salinity, drought, low temperature, and UV light, which can cause damage to lipids, proteins and DNA, reducing plants genome stability, growth, and productivity [1, 2]. Plants respond to these stresses via the cascades of molecular networks and changes in expression profiles of diverse classes of genes, including those involved in nucleic acid metabolism, which are induced in response to the DNA damage by activating a network of DNA repair enzymes including helicases [3–5]. RNA helicases, which catalyze the unwinding of energetically stable duplex RNA secondary structures in an ATP-dependent manner, thereby have been implicated in every aspect of RNA metabolism, including nuclear transcription, pre-mRNA splicing, ribosome biogenesis, RNA transport, translation initiation, RNA degradation, and organelle gene expression [6, 7]. Whereas DNA helicases unwind duplex DNA and are involved in replication, repair, recombination, and transcription [8].

The majority of RNA helicases belong to the superfamily 2 (SF2) subclass, characterized by sequence homology within the helicase domain that contains eight or nine conserved motifs. The SF2 consists of three subfamilies, termed DEAD, DEAH and DExH/D, based on variations within motif II of the proteins [9, 10]. The DEAD-box RNA helicases are by far the largest family of RNA helicases, and they are found in all of the organisms from prokaryotes to eukaryotes, and have been shown to be involved in almost every aspect of RNA metabolism [11, 12]. In addition, DEAD-box RNA helicases are also prominent candidates for RNA chaperones because they can use energy derived from ATP hydrolysis to disrupt misfolded RNA structures and promote correct folding, potentially playing roles in diverse cellular functions [9]. So far, biochemical activities characteristic of RNA helicases, namely RNA-dependent RNA unwinding and ATPase were only demonstrated for a small proportion of the RNA helicase. For example, the redox-regulated cyanobacterial helicase, CrhR, catalyzes annealing of complementary ssRNA (single-stranded RNA) into dsRNA (double-stranded RNA) and combines the helicase and annealing activities to promote RNA-strand exchange [13].

In plants, the comprehensive analysis and classification of the RNA helicase gene family in *Arabidopsis* and rice, which contain 113 and 115 RNA helicase genes, respectively, has been reported recently [14]. Presently, limited reports have emerged indicating that DEAD-box RNA helicase expression or activity is regulated not only with respect to participation in those housekeeping processes mentioned above, but also in plant growth and development, and in response to biotic and abiotic stresses [5, 15–17]. For example, the tobacco *VDL* gene encodes a plastid DEAD-box protein involved in chloroplast differentiation and plant morphogenesis as its recessive mutant *vdl* showed variegated leaves and abnormal roots and flowers [18]. In rice, the *OsBIRH1* gene encodes a DEAD-box RNA helicase, ectopic expression of *OsBIRH1* in *Arabidopsis* showed increased tolerance to pathogen and oxidative stresses [17]. The *Arabidopsis LOS4* RNA helicase was showed to be a cold stress-regulated gene, which was proved as an early regulator of CBF transcription factor expression in response to chilling [19, 20]. In addition, overexpression of *AtRH25* results in enhanced freezing tolerance in transgenic *Arabidopsis* [21]. On the contrary, mutations in *STRS1* (*STRESS RESPONSE SUPPRESSOR 1*) and *STRS2* which encode DEAD-box RNA helicases, cause increased expression of stress-responsive transcriptional activators and abiotic stress tolerance, suggesting that both STRS1 and STRS2 function as negative regulators [16]. Despite the involvement of DEAD-box RNA helicases in many diverse biological processes, the actual biological, biochemical, and molecular function of most plant DEAD-box proteins still remain to be further elucidated.

Tomato (*Solanum lycopersicum*) is one of the most widely produced and versatile vegetable crops, and it is also considered as one of the best-characterized plant systems employed in genetic and developmental studies whose genome has been fully sequenced [22, 23]. Although a genome-wide analysis of the RNA helicase gene family has identified 42 DEAD-box genes, 36 DEAH-box genes, and 52 DExD/H-box genes in tomato [24], none of the DEAD-box RNA helicase genes was well characterized in tomato so far. To explore the exact roles of tomato DEAD-box helicases, in the present study, two putative DEAD-box RNA helicase genes, termed *SlDEAD30* and *SlDEAD31*, were cloned and characterized from tomato. The overexpression of *SlDEAD31* led to enhanced salt and drought tolerance, and upregulated expression of stress-related genes. Our results suggest that *SlDEAD31* acts as a positive regulator in defence responses against abiotic stress, and may hold promising applications in the engineering of salt- and drought-tolerant plants.

## Results

### Sequence Analysis of *SlDEAD30* and *SlDEAD31* Genes

**Fig 1:**
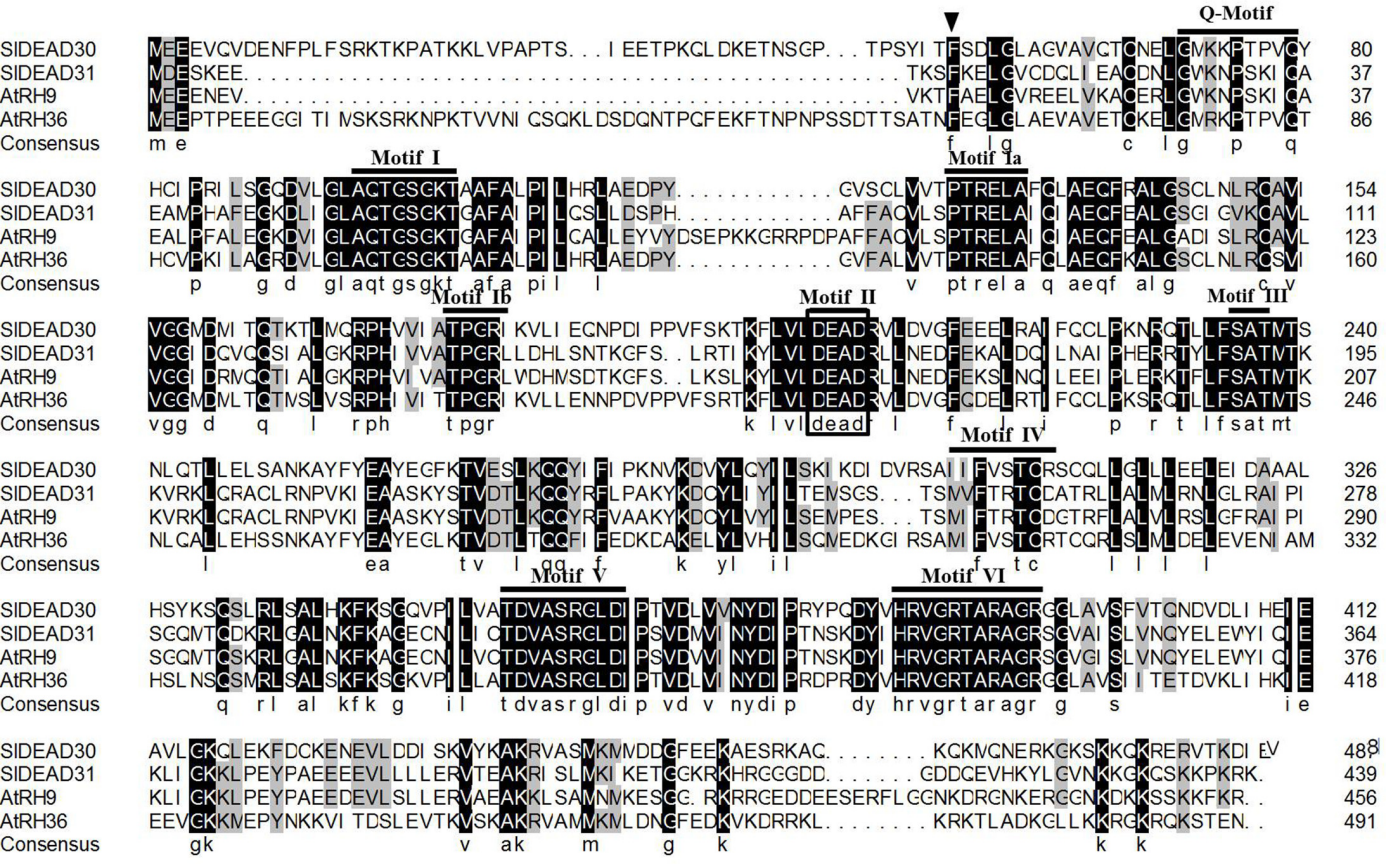
Multiple sequence alignment of the amino acid sequences of SlDEAD30, SlDEAD31, AtRH9, and AtRH36 proteins. Identical or similar amino acids are shaded in black and grey, respectively. The locations of the nine conserved helicase motifs are indicated on top of the sequences and DEAD domains are shown in the box. The conserved phenylalanine residue is indicated by an inverted triangle. SlDEAD30 (KJ739798), SlDEAD31 (KJ713393), AtRH9 (NM_125492), and AtRH36 (NM_101494).

Sequence analysis showed that *SlDEAD30* and *SlDEAD31* encode polypeptides consisting of 488 and 439 amino acids, and share 50.6% and 42.9% identity at the nucleotide level and amino acid level, respectively. In addition, two typical conserved domains: the DEAD-box helicase superfamily domain and the helicase C-terminal domain, as well as nine conserved helicase motifs, which are common characteristics of DEAD-box RNA helicases, are all present in these two SlDEAD proteins (S1 and S2 Figs). Generally, these motifs are functional in RNA substrate binding and ATP binding or exhibit ATPase activity [10]. To further analyze the conserved helicase motifs of SlDEAD30 and SlDEAD31 proteins, the amino acid sequences of two previously reported DEAD-box proteins, AtRH9 and AtRH36 from *Arabidopsis*, which show a high sequence similarity with SlDEAD30 and SlDEAD31, were analyzed for alignment. SlDEAD30 protein shares a close relationship with AtRH36 (66.4% identity, S1 Table), which is essential for *Arabidopsis* female gametogenesis [25], whereas SlDEAD31 is much closer related to AtRH9 (79.9% identity, S1 Table), which has an important role in cold tolerance in *Arabidopsis* [21]. The results show that all the nine conserved helicase motifs are present in the four DEAD-box proteins, Q-motif and motif IV are relatively diverse, while the other motifs are highly conserved (Fig 1).

**Fig 2:**
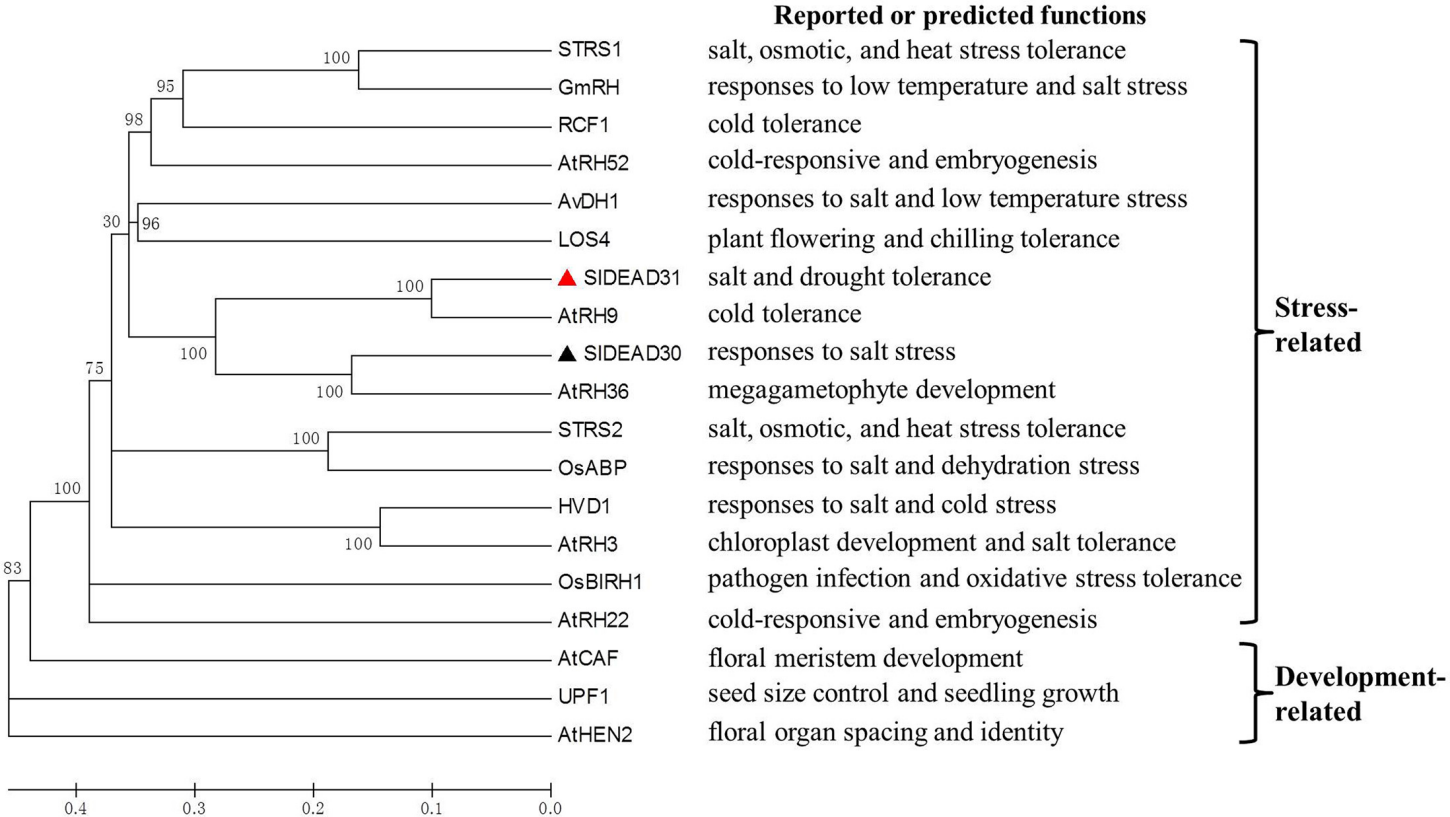
Phylogenetic tree and reported or predicted functions of SlDEAD30, SlDEAD31, and other known plant DEAD-box proteins. The phylogenetic tree analysis was constructed with MEGA 5.2 software using the neighbor-joining method and the following parameters: bootstrap analysis of 1000 replicates, poisson model and pairwise deletion. The numbers at the nodes indicate the bootstrap values. SlDEAD30 and SlDEAD31 are marked with a black triangle and a red triangle, respectively. Accession numbers and corresponding references for the proteins used are as follows: *Solanum lycopersicum*: SlDEAD30, KJ739798; SlDEAD31, KJ713393. *Arabidopsis thaliana*: AtRH3, NM_001036866 [28]; AtRH9, NM_125492 [21]; AtRH22, NM_104691 [26]; AtRH36, NM_101494 [25]; AtRH52, NM_115719 [26]; STRS1, AY080680 [16]; STRS2, AY035114 [16]; LOS4, BT002444 [19]; RCF1, BT002030 [54]; AtCAF, AF187317 [27]; AtHEN2, AY050658 [55]. UPF1, AF484122 [56]. *Oryza sativa*: OsBIRH1, Q0DVX2 [17]; OsABP, LOC_Os06g33520 [57]; *Hordeum vulgare*: HVD1, AB164680 [58]; *Glycine max*: GmRH, FJ462142 [59]. *Apocynum venetum*: AvDH1, EU145588 [15].

To investigate the divergence of SlDEAD30 and SlDEAD31 proteins with other plant DEAD-box proteins during evolution, a phylogenetic tree was constructed using the amino acid sequences of SlDEAD30, SlDEAD31, and 17 previously published plant DEAD-box proteins with known functions. These proteins are putatively divided into two distinct subgroups, a stress-related subgroup and a development-related subgroup based on their relatives’ function and the phylogenetic tree. SlDEAD30 and SlDEAD31 proteins are present in the stress-related subgroup (Fig 2). In addition, the phylogenetic analysis also revealed that SlDEAD30 and SlDEAD31 proteins share a close relationship with the AtRH36 and AtRH9, respectively, suggesting that they may carry out similar function. Besides, the possible functions of SlDEAD30, SlDEAD31, and the other previously reported plant DEAD-box RNA helicases are also presented (Fig 2).

### Organ-Specific Expression of *SlDEAD30* and *SlDEAD31* Genes in Wild-Type Tomato

**Fig 3:**
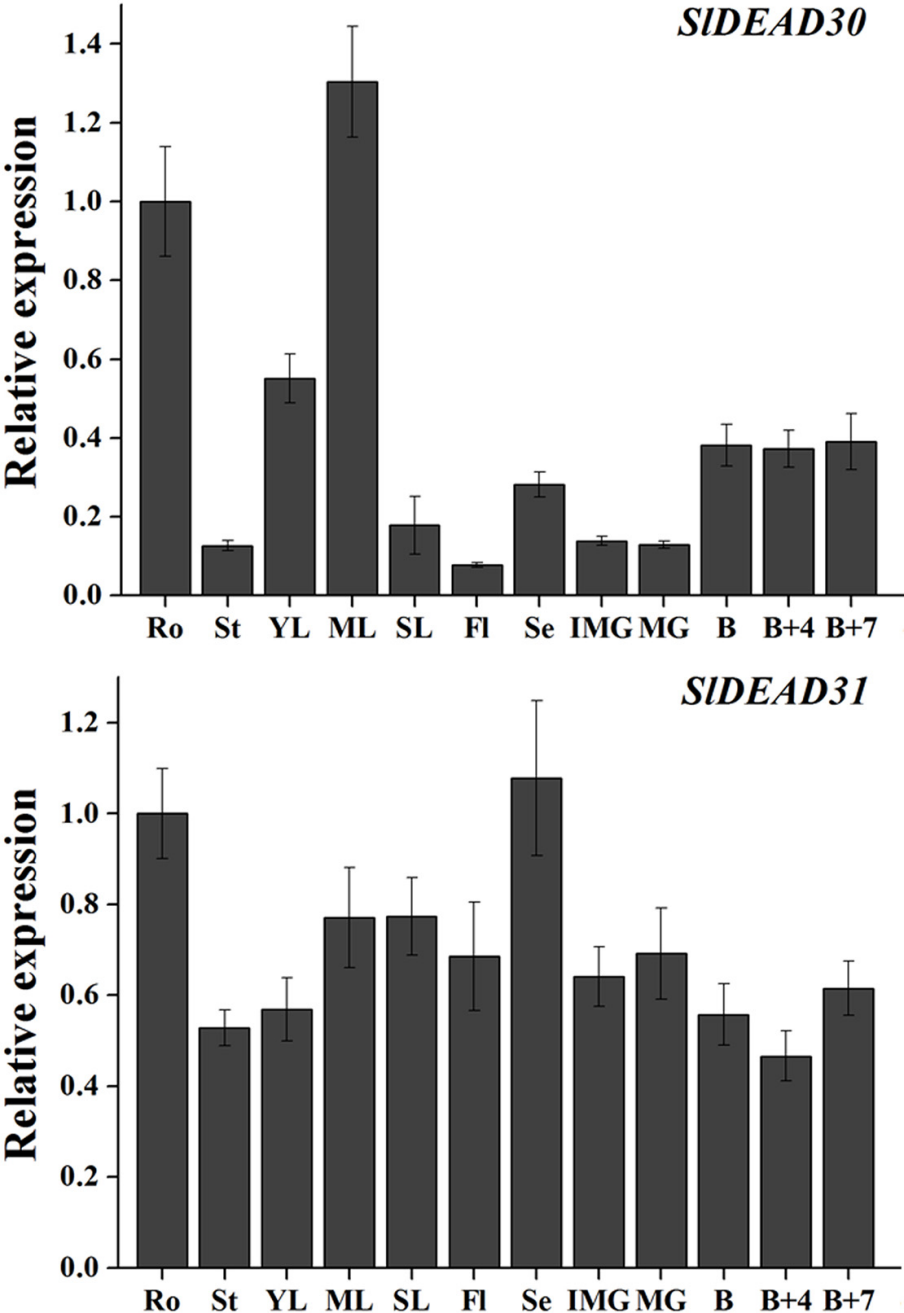
Expression profiles of *SlDEAD30* and *SlDEAD31* genes in different tissues of wild-type tomato. Ro, roots; St, stems; Yl, young leaves; Ml, mature leaves; Sl, senescent leaves; Fl, flowers; Se, sepals; IMG, immature green fruits; MG, mature green fruits; B, breaker fruits; B+4, 4 day after breaker stage; B+7, 7 day after breaker stage. The relative expression levels were normalized to 1 in the roots. Bars represent mean relative expression values ± SE (*n* = 3).

Tissue specificity may be associated with specific biological functions. Previous reports have demonstrated that a range of DEAD-box RNA helicases are involved in regulating the essential steps in plant growth and development, such as morphogenesis and embryogenesis [18, 26], floral meristems [27], and chloroplast development [28]. The results showed that the expression of *SlDEAD30* was relatively higher in roots and mature leaves, whereas moderate to weak signals of its expression were detected in other tissues such as stems, flowers, and fruits. *SlDEAD31* mRNAs were constantly expressed in all the organs tested (Fig 3).

### The Expression of *SlDEAD30* and *SlDEAD31* Genes in Response to Various Abiotic Stresses

**Fig 4:**
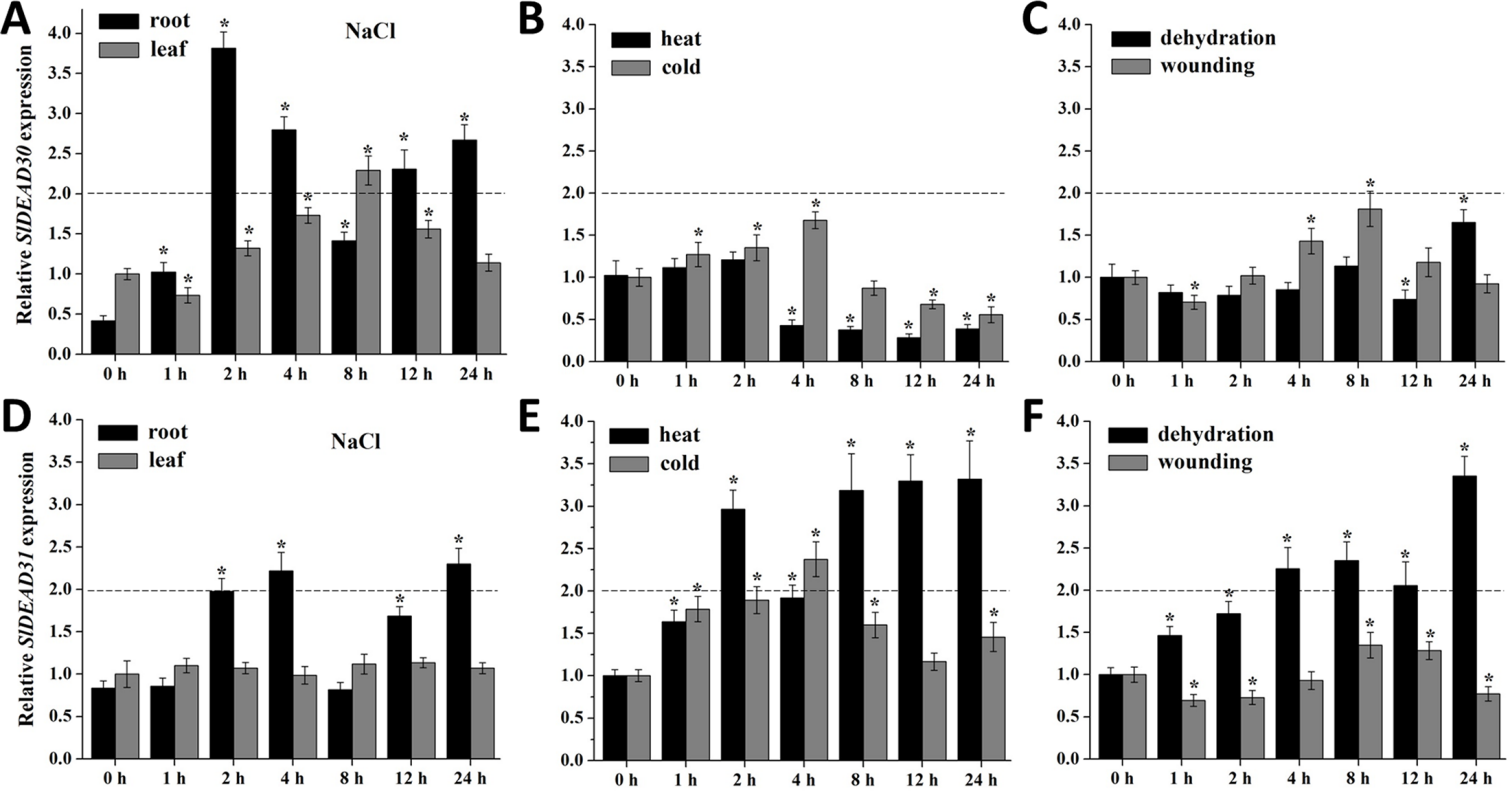
Expression patterns of *SlDEAD30* and *SlDEAD31* genes under various stress treatments including NaCl (200 mM, A, D), heat (40°C) and cold (4°C, B, E), dehydration, and wounding (C, F). Gene expression was detected by RT-PCR using total RNA from leaves or roots of 35-day-old tomato plants. Bars represent mean relative expression values ± SE (*n* = 3). Values are presented relative to untreated plants (0 h). Asterisks indicate a significant difference (P<0.05) between untreated and treated plants. The twofold threshold is indicated by a dotted line.

To speculate on the physiological and functional relevance of *SlDEAD30* and *SlDEAD31* genes, we detected their expression profiles under various abiotic stresses by quantitative RT-PCR. We found that their expression was not affected by circadian rhythm under the normal growth condition (S3 Fig). *SlDEAD30* transcripts were induced apparently in roots after NaCl stress compared with controls, and to a lesser degree in leaves (Fig 4A). *SlDEAD31* expression was also induced by NaCl in roots, whereas remained unchanged in leaves (Fig 4D). The results indicate that the expression of both *SlDEAD30* and *SlDEAD31* was induced mainly in roots but not in leaves under NaCl stress. When treated with heat and cold, *SlDEAD30* mRNA was not increased by cold, while down-regulated by heat (Fig 4B). In contrast, *SlDEAD31* expression was induced by both stresses (Fig 4E). In addition, the expression of *SlDEAD30* was not induced by dehydration and wounding, while *SlDEAD31* mRNA was induced by dehydration almost by a gradual increase (Fig 4C and 4F). The induction of *SlDEAD30* and *SlDEAD31* genes by abiotic stress prompted us to check their promoter sequences by searching against the promoter database PLACE. The results show that their promoter sequences indeed contain putative stress response-related cis-elements, such as MYB, MYC, and WRKY recognition sites (S2 Table).

### Overexpression of *SlDEAD31* in Tomato Plants Significantly Improves Salt Tolerance

**Fig 5:**
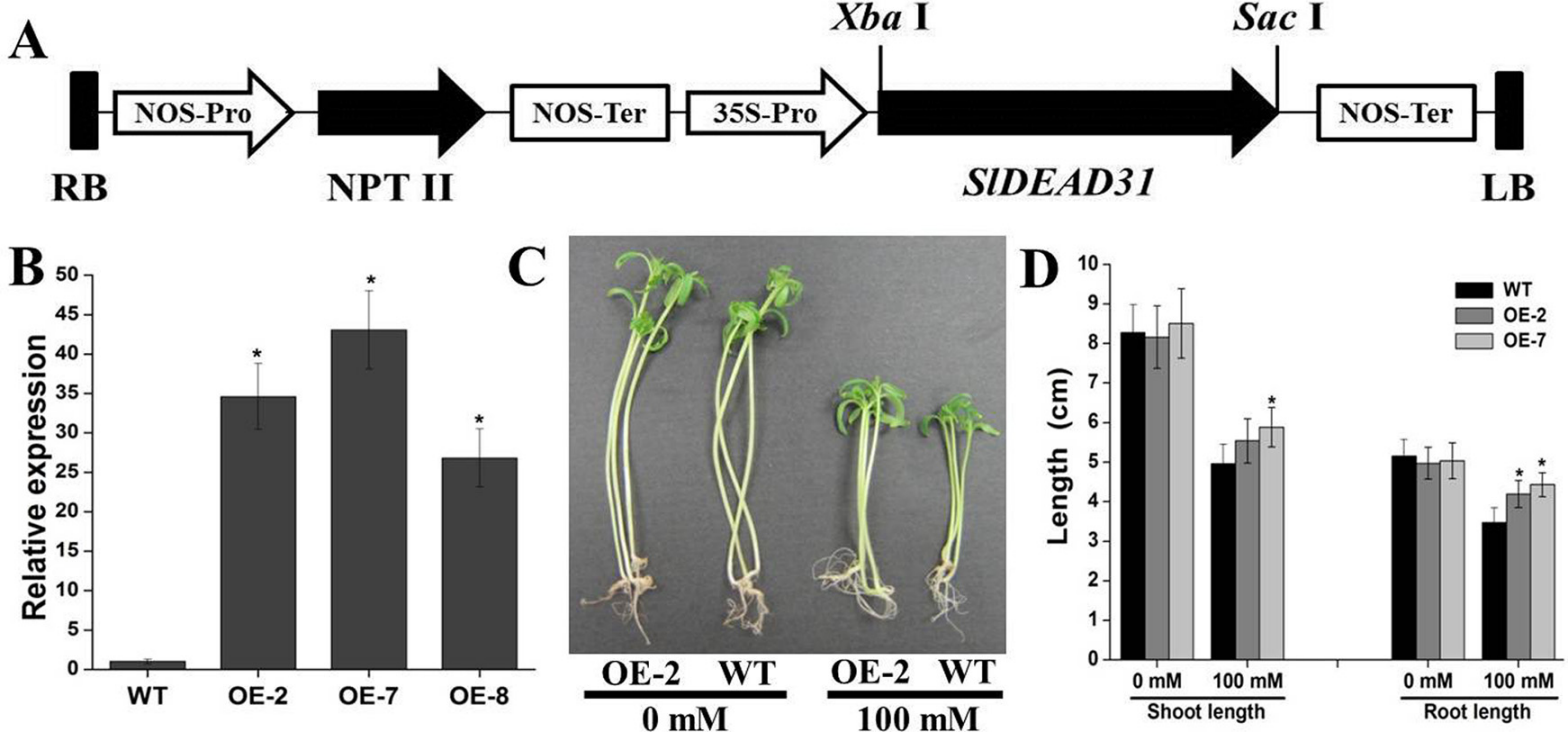
Salt sensitivity of *SlDEAD31* overexpressing plants at postgermination stage. (A) *SlDEAD31*-overexpressing vector. RB, right border; Pro, promoter; Ter, terminator; LB, left border. (B) Quantitative RT-PCR analysis of *SlDEAD31* expression in the leaves of WT and transgenic lines OE-2, OE-7, and OE-8. Values represent the means ± SE (*n* = 3). Asterisks indicate a significant difference from WT (p<0.05). (C) and (D) Representative growth performance (C) and shoot and root length (D) of WT and *SlDEAD31* overexpressing plants in MS medium containing 0 and 100 mM NaCl at postgermination stage. The shoot and root length of seedlings (n≥20 each) were counted after growth for 10 d. Values represent the means ± SE (*n* = 3). Asterisks indicate a significant difference from WT (p<0.05).

Taking into account that only *SlDEAD31* expression was induced by various stresses, therefore, to further analyze its biological function, we generated transgenic tomato plants overexpressing *SlDEAD31* (Fig 5A). A total of 7 independent *SlDEAD31*-overexpressing transgenic lines were obtained. Besides, we also attempted to silence *SlDEAD31* gene in stably transformed tomato plants by RNAi (RNA interference) using the constitutive 35S promoter, but this failed three times. Therefore, only *SlDEAD31*-overexpressing transgenic lines were used for further studies. Quantitative RT-PCR analysis showed that the relative mRNA level of *SlDEAD31* in three overexpression transgenic lines (OE-2, OE-7, and OE-8) increased by 34.6, 43.1, and 26.8 times compared with WT plants, respectively (Fig 5B).

Salt sensitivity in tomato is most evident during seed germination and subsequent seedling growth [29]. As described above, the induction of *SlDEAD31* expression by NaCl suggested that it might be involved in the tolerance to salt stress (Fig 4D). To confirm this, salt tolerance performance of the transgenic tomato seedlings was tested at the post-germination stage. WT and transgenic tomato seeds of consistent germination were transferred to medium containing 0 and 100 mM NaCl. In the presence of 100 mM NaCl, plant growth was inhibited apparently in both WT and overexpressing tomato seedlings, whereas the average length of roots of WT seedlings were shorter than that of transgenic plants, while the difference of shoot length was not that significant. No discernible difference was observed for seedlings grown in the control medium (Fig 5C and 5D), indicating that the salt-caused retarded growth on WT plants was more severe than on transgenic plants.

**Fig 6:**
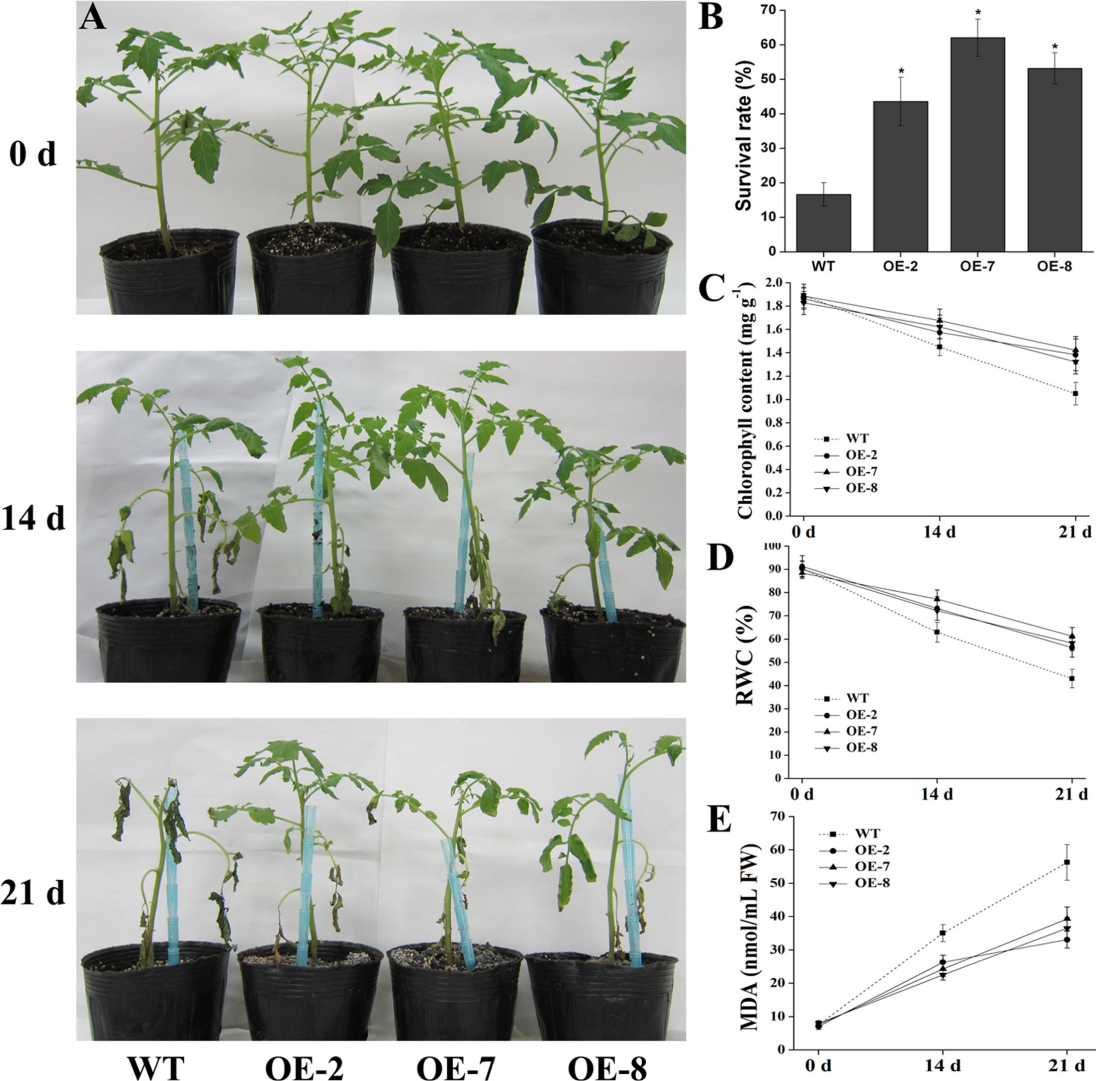
Salt stress tolerance of *SlDEAD31-*overexpressing plants. (A) Growth characteristics of WT and transgenic tomato plants at 0, 14 and 21 d after salt stress. 8-week-old plants were irrigated with 200 mL 400 mM NaCl every 72 h from the bottom of the pots. Representative plants are shown. (B) Survival rate of the plants shown after 21 d of salt treatment and 5 d of recovery. Each bar represents an average of eight plants ± SE (n = 3). Asterisks indicate a significant difference between WT and transgenic lines (*P*<0.05). (C)-(E) Comparisons of chlorophyll content (C), relative water content (RWC, D), and malondialdehyde (MDA) content (E) of WT and transgenic plants at 0, 14 and 21 d after salt stress. Data represent the means ± SE (*n* = 3).

To further evaluate the performance of *SlDEAD31*-overexpressing plants under salt stress in soil, 8-week-old plants of WT and transgenic lines were irrigated with water containing 400 mM NaCl (200 mL) every 72 h. Under normal growth conditions, the transgenic plants showed no abnormal morphological phenotype compared with WT plants (Fig 6A). However, WT plants showed more notorious and quick damage signs during all the time course of the salt assay. NaCl-induced symptoms were observable 14 d after salt stress with chlorosis and wilting of lower leaves in both WT and transgenic plants, whereas the transgenic lines displayed apparently less wilting and necrosis, and most of the leaves remained vigorous. After 21 d, all the leaves of WT plants showed severe necrosis and wilting, and some were dead, whereas the upper leaves of transgenic plants remained green and vigorous (Fig 6A). Besides, most of the WT plants were dead 5 d after stopping the treatment, whereas 43–62% of the transgenic plants survived (Fig 6B).

Salt damage was evaluated by measuring chlorophyll content, relative water content (RWC) and malondialdehyde (MDA) content, which are typical physiological parameters for evaluating abiotic stress tolerance in crop plants. Under normal conditions, these physiological parameters were similar for transgenic and WT plants (Fig 6C–6E). Upon exposure to salt stress, there were obvious reductions in chlorophyll content in the leaves of both WT and transgenic tomato plants. WT plants lost about 23 and 43% of the chlorophyll at 14 and 21 d after salt assay, respectively. However, in *SlDEAD31*-overexpressing plants this reduction was about 11–16 and 24–29%, respectively (Fig 6C). RWC in transgenic plants decreased to 72–77% of the normal levels at 14 d, and to 56–61% at 21 d post-treatment. Whereas this parameter in WT plants went down to about 63 and 43% at 14 and 21 d post-treatment, respectively (Fig 6D). The MDA content, which is an indicator of lipid peroxidation, was increased by the salt treatment, but the degree of increase in transgenic plants was lower than in WT plants (Fig 6E), suggesting that overexpression of *SlDEAD31* induces potential antioxidative processes preventing significant membrane damage.

### Stress-Related Genes Are Upregulated in *SlDEAD31*-Overexpressing Tomato Plants

**Fig 7:**
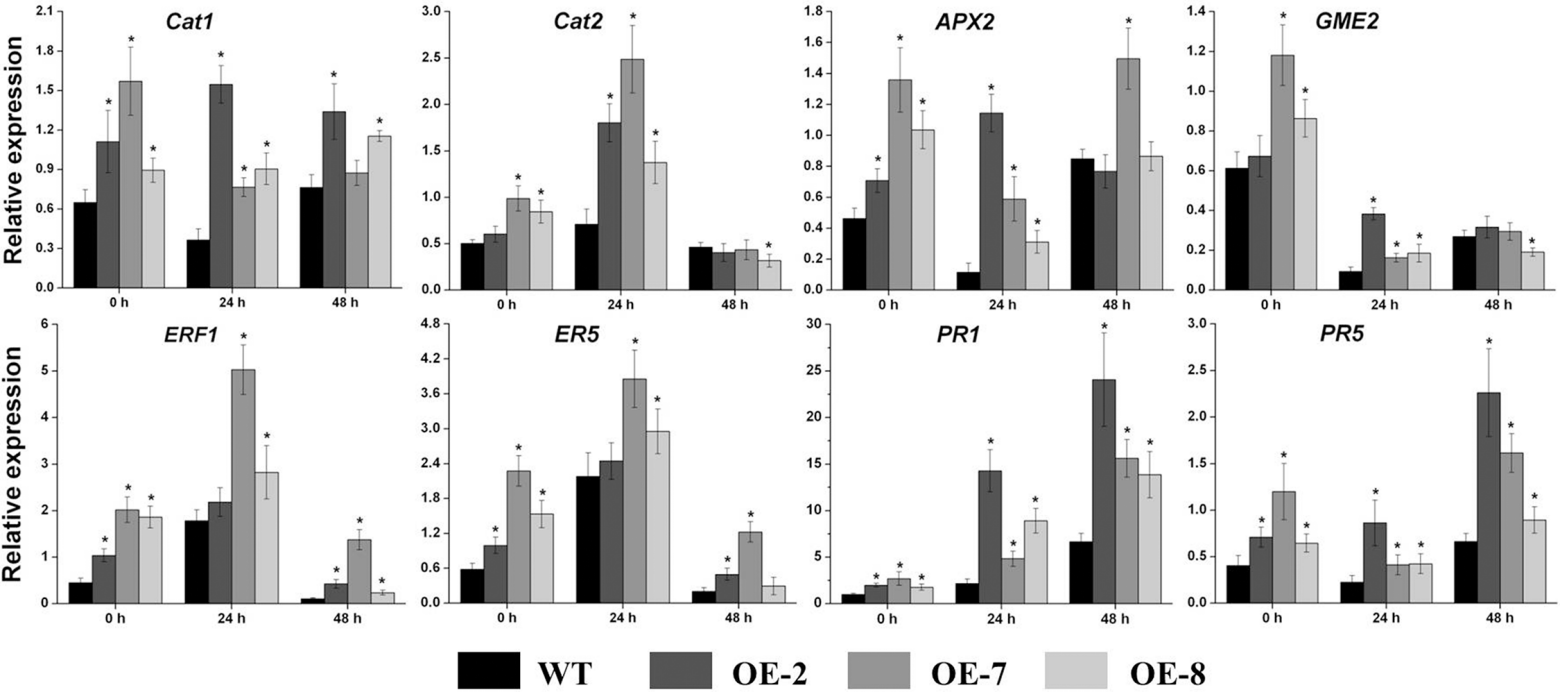
The relative expression levels of biotic and abiotic stress-related genes of WT and *SlDEAD31-*overexpressing plants under normal and salt stress conditions. 8-week-old plants were irrigated with water containing 400 mm NaCl (200 mL) from the bottoms of the pots, then root samples were harvested after 24 and 48 h. Seedlings harvested before the salt stress were used as controls. Bars represent mean relative expression values ± SE (*n* = 3). Asterisks indicate a significant difference (P<0.05) between WT and transgenic plants.

To gain further information concerning the increased salt tolerance phenotype observed in *SlDEAD31*-overexpressing tomato plants, transcripts of previously reported stress-related genes were utilized for comparative analysis between WT and transgenic tomato roots under normal and salt-stressed conditions. The key ascorbic acid (AsA) synthetase gene *GME2* [30]; two catalase (CAT) genes, *Cat1* and *Cat2* [31]; an ascorbate peroxidase (APX) gene, *APX2* [32]; an ethylene-responsive *LEA* gene (*ER5*) [33]; the ethylene-responsive factor ERF1 [34], and two pathogenesis-related (PR) genes, *PR1* and *PR5* [35] were selected (Fig 7). Based on the reported literature, these stress tolerance genes are involved in the regulation of biotic and abiotic stress responses (such as *PR1*, *PR5*, *ER5*, and *ERF1*), production of osmolytes (such as *GME2*), detoxification and redox homeostasis (such as *Cat1*, *Cat2*, and *APX2*). Quantitative RT-PCR analysis showed that the transcripts of *Cat1*, *ERF1*, *ER5*, *PR1*, and *PR5* were significantly elevated in almost all the overexpression lines under both normal and salt-stressed conditions, although the fold changes showed some variation for some genes among the three transgenic lines (Fig 7). Relatively higher expression levels of *Cat2*, *APX2*, and *GME2* were detected mainly under normal conditions and at 24 h following salt stress in overexpression lines. The transcripts of these stress-related genes were also analyzed in the leaves of WT and transgenic plants, the results showed that their induction levels were less significant than in roots, as expected (taking into account that the expression of *SlDEAD31* was only induced in roots by NaCl stress, Fig 4D, S4 Fig).

### Overexpression of *SlDEAD31* in Tomato Plants Slightly Improves Drought Resistance

**Fig 8:**
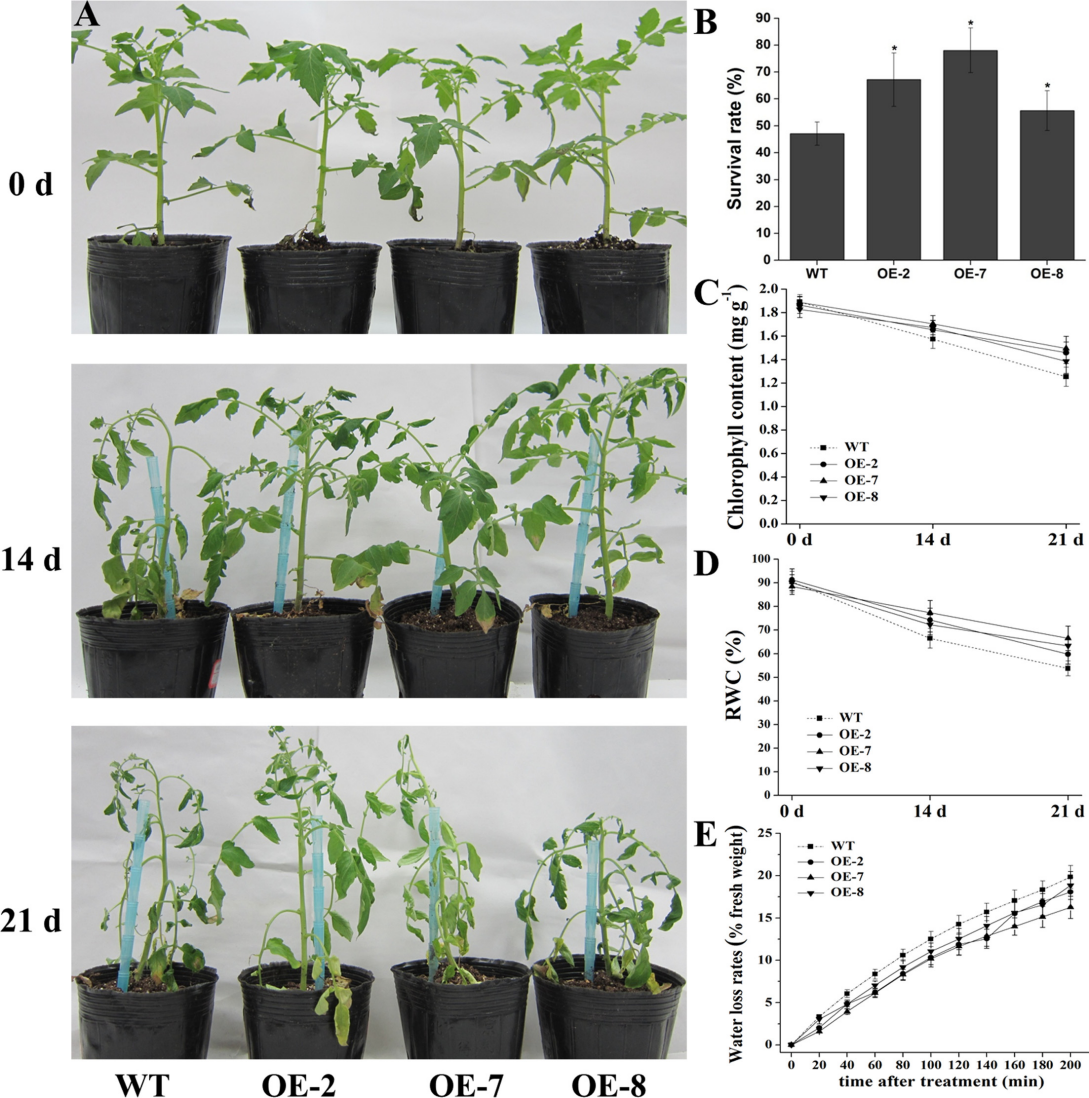
Drought tolerance testing of *SlDEAD31-*overexpressing plants. A water withholding assay was performed with 8-week-old plants for up to 21 d followed by a recovery period of 5 d. (A) Representative phenotypes of wild-type and *SlDEAD31* overexpression plants at 0, 14 and 21 d after initiation of drought assay. (B) Survival rates of WT and transgenic plants after rewatering for 5 d following 21 d of drought assay. Each bar represents an average of eight plants ± SE. Asterisks indicate a significant difference from WT (p < 0.05). (C) and (D) Comparisons of chlorophyll content (C) and relative water content (RWC, D) of WT and transgenic plants at 0, 14 and 21 d after drought treatment. Values represent the means ± SE (*n* = 3). (E) Water loss rates of WT and transgenic plants. Similar leaves were excised and weighed at the indicated times. Water loss was calculated as the percentage of initial fresh weight. At least 15 leaves of similar developmental stages were excised and weighed at different times after the detachment. The data represent the means ± SE (*n* = 3).

To further test the regulatory effects of *SlDEAD31* on drought resistance, a drought assay was performed by withholding water for 21 d in soil and then physiological parameters were measured. The leaves of WT plants started to show typical drought-induced symptoms after 14 d of drought stress, such as leaf rolling and wilting with a concomitant loss of chlorophyll (Fig 8A). Whereas the transgenic plants showed delayed leaf-rolling compared with the WT, only slight symptoms of drought-induced damage were observed, and most of the leaves remained vigorous. Only slight differences were observed on the 21th day after drought stress, almost all the leaves of WT plants displayed severe wilting and curling, while slightly less withered and rolled leaves were observed in transgenic plants (Fig 8A). In addition, after rewatering the plants for 5 d, 55–78% of *SlDEAD31* transgenic plants recovered from the drought stress, while about 47% of the WT plants recovered (Fig 8B).

To further verify the drought tolerance phenotype, the chlorophyll and RWC values of WT and transgenic plants were monitored in leaves during the drought assay. Apparent chlorophyll degradation was observed in both WT and transgenic plants under drought stress, while the reduction in WT was higher than that in transgenic plants both at 14 and 21 d of the drought assay (Fig 8C). RWC in WT plants decreased to about 67 and 54% at 14 and 21 d post-treatment, respectively. Whereas in transgenic lines, this parameter went down to about 73–79% at 14 d and to 60–67% at 21 d following the same drought stress, respectively (Fig 8D). In addition, the water loss rate of detached leaves, which is also an important physiological parameter to investigate plants’ drought tolerance, was slightly lower in *SlDEAD31*-overexpressing plants compared to the WT at each dehydration time point (Fig 8E).

## Discussion

Agricultural productivity is subjected to increasing environmental constraints, particularly to drought and salt stress due to their high magnitude of impact and wide distribution. The *Solanaceae* family, which includes tomato, potato, pepper, and tobacco, represents one of the most valuable families of vegetable crops. Cultivated tomatoes are susceptible to a wide range of environmental stresses, for example, most tomato cultivars being moderately sensitive to drought and salt stress during seed germination, seedling emergence, vegetative growth, and reproduction, thus fail to produce high yields in a fragile ecosystem [36]. Bioengineering stress-signaling pathways to develop stress-tolerant crops is one of the major goals of agricultural research. The involvement and significance of RNA helicases in response to biotic and abiotic stresses have only recently begun to emerge, and Vashisht and Tuteja (5] suggested that stress-responsive DEAD-box helicases have uncovered a new pathway to engineer plant stress tolerance. In the present study, we characterized two tomato DEAD-box genes, *SlDEAD30* and *SlDEAD31*, which each encodes a putative DEAD-box RNA helicase with all the conserved domains and motifs that are characteristic of DEAD-box proteins. Our phylogenetic analysis showed that they cluster together with stress-related DEAD-box proteins, which was further supported by the induction of *SlDEAD30* and *SlDEAD31* by multiple abiotic stresses (Figs 2 and 4), and that overexpression of *SlDEAD31* significantly enhanced salt tolerance and slightly improved drought resistance. Besides, MYB, MYC, and WRKY transcription factors have been suggested to be involved in responses and adaptation to biotic and/or abiotic stress in plants [37, 38]. Thus, the presence of these stress responsive cis-acting elements also suggests critical roles of these two genes in stress tolerance. Moreover, phylogenetic analyse indicated that a cross-talk may exist between stress response and plant development in DEAD-box RNA helicases. For example, in the stress-related subgroup, the *Arabidopsis LOS4* plays a role in chilling tolerance linked with other developmental processes such as flowering or vernalization [19, 20]. *Arabidopsis RH3* gene plays a critical role in chloroplast development, while *rh3* mutant was more sensitive to NaCl stress [28]. Similarly, the high or constant expression of *SlDEAD30* and *SlDEAD31* in specific tissues suggest that they may also have a specialized function in plant growth and/or reproductive development, while further studies are needed.

Evidence that *SlDEAD31* plays important roles in salt and drought tolerance, as demonstrated by its inducible expression by multiple abiotic stress treatments, including NaCl and dehydration, as well as by phenotypes, physiological indices, and significantly enhanced expression of multiple stress-related genes in the *SlDEAD31*-overexpressing tomato plants, was presented. Similarly, there are several examples in which similar effects have been demonstrated through the overexpression of stress-responsive DEAD-box helicase genes that can improve the stress tolerance of transgenic plants. For example, *OsBIRH1*-overexpressing transgenic rice showed an enhanced disease resistance and oxidative stress tolerance [17]. *PDH45* was proven to be induced by various abiotic stresses including NaCl, and its overexpression in rice confers salinity tolerance and improved physiological traits [39].

The establishment of seedlings at early growth stages, which is one of the most important determinants for crop production, is severely affected by soil salinity. Therefore, vigorous early growth under salty soils is preferred. In our present study, we observed a better growth of *SlDEAD31*-transgenic lines compared to WT plants (Fig 5C and 5D), indicating enhanced salt tolerance during this sensitive and critical stage. In addition, environmental stresses often cause physiological changes in plants. Physiological indices, including chlorophyll content, RWC and MDA, are typical and effective physiological parameters for evaluating abiotic stress tolerance and resistance in crop plants. Chlorophyll content, expressing the retention of chlorophyll, is taken as an index of the damage done to the photosynthetic apparatus. Leaf RWC, expressing the relative amount of water present in the plant tissues, is a useful indicator of plant water balance. Adverse stress conditions often generate reactive oxygen species (ROS), thus inducing oxidative damage to macromolecules such as membrane lipids. The content of MDA can serve as an indicator of oxidative damage in the cell [40]. Our results showed that both the RWC and chlorophyll content of transgenic lines were higher than in WT plants under salt- and drought-stress conditions (Figs 6 and 8), suggesting that *SlDEAD31*-overexpressing plants had more robust photosynthetic capabilities and better water balance than the WT. In addition, transgenic tomato plants showed lower MDA content than the WT plants under salt stress (Fig 6E), which may result from an enhanced capacity for scavenging ROS. Water loss rate is an important parameter of plants under water-deficit conditions and has been proposed as an indicator of water status [41]. Therefore, the slightly enhanced drought tolerance of transgenic plants could be attributed, at least in part, to their lower transpiration rates (Fig 8E). In addition, *SlDEAD31*-overexpressing plants showed significantly higher survival rates than WT plants under both stresses (Figs 6B and 8B), further supporting the usefulness of *SlDEAD31* in improving salt and drought resistance in tomato.

The expression of multiple abiotic stress-related genes was significantly upregulated in the *SlDEAD31*-overexpressing tomato plants (Fig 7), something considered to be advantageous for the resistance to abiotic stress, also indicating the protective role of *SlDEAD31* against environmental stresses. The expression of stress-related genes results in the accumulation of metabolites and the alteration of biochemical and physiological pathways that are vital for the plant adaptation to adverse stress conditions. Our results are in a good agreement with previous report, in which enhanced tolerance to various stresses is mainly attributed to increased expression of biotic and abiotic stress-related genes in the *OsBIRH1-*overexpressing plants [17]. In plants, biotic and abiotic stresses can trigger the generation of ROS, which include hydrogen peroxide (H_2_O_2_) that can oxidize and damage cellular components [42]. The ROS defense mechanisms involve the synthesis of antioxidant compounds (such as AsA) and antioxidant enzymes (such as CAT1/2 and APX2). An increase in AsA content can confer tolerance to various stresses including salt, ozone, and chilling [30]. The upregulated expression of *GME2* was detected in the transgenic plants suggesting an elevated AsA content, and thus conferring tolerance to salt stress in *SlDEAD31*-overexpressing plants. In this context, CAT and APX can detoxify ROS by converting H_2_O_2_ to H_2_O in plant cells under oxidative stress [31, 32]. Therefore, the enhanced tolerance observed in transgenic tomato plants might be due to the upregulation of *APX2* and *Cat1*/*2*, and thus an increased protection against ROS-induced damages. In addition, a significant upregulation in *ERF1* expression was observed in transgenic plants. It has been shown that overexpression of *ERF1* provides improved drought tolerance in transgenic tomato [34]. We have reported previously that overexpression of *SlERF5* in tomato promotes adaptation to drought and salt tolerance [43]. Furthermore, *ER5*, an ethylene-responsive *LEA* gene confers desiccation tolerance [33], was also increased in transgenic plants, indicating a less damage of the transgenic lines under abiotic stress. Interestingly, transcripts encoding pathogenesis-related proteins, *PR1* and *PR5*, were also upregulated in transgenic lines. Similarly, they were also elevated in the O*sBIRH1*-overexpressing *Arabidopsis* plants [17]. Thus our results imply that *SlDEAD31*, besides having a role in plant adaptation to abiotic stress, may also integrates signals derived from both biotic and abiotic stresses in tomato, although this remains to be elucidated in detail. Collectively, our data indicate that biotic or abiotic stresses can induce the expression of *SlDEAD31*, leading to the upregulation of transcripts encoding functional and regulatory proteins involved in antistress metabolism. Therefore, their consistent upregulated expression likely increases the expression of downstream genes, probably enhancing integrative tolerance to multiple stresses due to cross-talk between various environmental stresses, especially salt and drought [44, 45].

In conclusion, the present study provides a preliminary characterization of *SlDEAD30* and *SlDEAD31* genes in tomato, and suggests that stress-responsive *SlDEAD31* functions in positive modulation of abiotic stress tolerance. Presently, the functions of RNA helicases are still poorly understood in plants. Clearly, understanding the role of *SlDEAD31* will not only extend knowledge of the biological function of the DEAD-box RNA helicases, but will also provide insight into the significance of DEAD-box RNA helicase-mediated metabolism in regulation of plant defence responses. Although the detailed mechanisms underlying the functions of *SlDEAD31* remain to be completely revealed, for example, what role SlDEAD31 may play in plant growth and development, how SlDEAD31 protein regulates the expression of stress-related genes, and how SlDEAD31 operates different stress signaling pathways in tomato, the data we present here indicate that overexpression of *SlDEAD31* confers enhanced salt and drought stress tolerance of tomato. Thus, *SlDEAD31* may hold a promising application in the production of stress-tolerant plants.

## Materials and Methods

### Identification and Isolation of *SlDEAD* Genes

To identify DEAD-box genes in tomato, the protein sequences of AtRHs (RNA helicase genes) from *Arabidopsis* were used as driver sequences to search the database of Sol Genomics Network (SGN, http://solgenomics.net/), which is a clade-oriented database containing biological data for the *Solanaceae*. Different AtRHs were used to interrogate the database, and the returned results were compared to each other. Only the sequences that contain the DEAD-box helicases superfamily domains and all the nine conserved RNA helicase motifs were selected and used for further analyse. For the two gene predictions selected eventually, *SlDEAD30* and *SlDEAD31*, gene-specific primers (S3 Table) were designed for PCR to isolate the cDNA, and their nucleotide and deduced amino acid sequence data have been deposited in GenBank under the accession numbers KJ739798 and KJ713393, respectively.

### DNA and Protein Sequence Analysis

The translation of SlDEAD genes using the ExPASy translate tool, grand average of hydropathicity (GRAVY) and theoretical molecular weight (Mw) were calculated using the ExPASy ProtParam tool (http://www.expasy.org/). Conserved structure domains were annotated according to the ScanProsite (http://prosite.expasy.org/scanprosite/). Multiple sequences alignment and phylogenetic tree were conducted using the DNAMAN 5.2.2 program and MEGA 5.2 software, respectively. To analyze putative cis-elements in the promoter regions of *SlDEAD30* and *SlDEAD31* genes, promoter sequences (1000 bp regions upstream the 5’ end of the predicted ORFs) of both genes were extracted from SGN database and searched against the promoter database PLACE (http://www.dna.affrc.go.jp/PLACE/index.html) [46].

### Plant Materials for Organ-Specific Expression

The wild-type (WT) tomato (*Solanum lycopersicum* Mill. cv. Ailsa Craig) seeds were germinated and the seedlings were grown in a greenhouse under sodium lights timed for 16 h days (27°C) and 8 h nights (19°C). For organ-specific expression profiling of *SlDEAD30* and *SlDEAD31* genes, the roots, stems, flowers, sepals, fruits, and leaves of similar age and position at different stages were collected. Flowers were tagged at anthesis and fruit development was recorded as days post-anthesis (DPA). The fruit ripening stages were divided into IMG (immature green, 28 DPA), MG (mature green, 35 DPA, full fruit expansion but no visible color change), B (breaker, fruit showing the first signs of ripening-associated color change from green to yellow), B+4 (4 d after breaker), and B+7 (7 d after breaker). All plant samples for the preparation of total RNA were taken at the same time each day, frozen in liquid nitrogen and stored at -80°C until required.

### Plant Stress Treatments

To verify the expression profiles of *SlDEAD30* and *SlDEAD31* genes under abiotic stress treatments, WT tomato seeds were germinated and the seedlings were grown in greenhouses. All the plant treatments were performed using potted 35 day-old tomato seedlings. In each case, individual plants were used for each treatment with three biological replicates. Untreated seedlings were used as controls, and all leaf or root samples were harvested at 1, 2, 4, 8, 12, 24 h after each treatment. Heat and cold treatments were conducted by incubating the seedlings at 4 and 40°C, respectively. Salinity treatment was applied by submerging the roots of seedlings in a solution with 200 mM NaCl, then root and leaf samples were collected. For dehydration, whole tomato seedlings were gently pulled out, washed carefully with water to remove the soil, and left on a piece of dry filter paper. For wounding, the leaves of tomato seedlings were cut with a razor blade into small pieces and left on wetted filter paper in sealed pots at 25 ±1°C [43, 47]. All harvested samples were immediately frozen in liquid nitrogen and stored at -80°C until RNA extraction.

### RNA Extraction and Quantitative RT-PCR Analysis

Total RNA was isolated from different plant tissues accordingly to the protocol of the Trizol reagent (Invitrogen, Shanghai, China). 2 μg of RNA were predigested with Dnase-I and reverse-transcribed using M-MLV reverse transcriptase (Promega, Beijing, China) with oligo(dT)_18_ primer. The quantitative RT-PCR reaction consisted of 5 μL 2× GoTaq qPCR Master Mix (Promega, Beijing, China), 0.25 μL 10 mM each primer, 1 μL cDNA and distilled water to a final volume of 10 μL. Quantitative RT-PCR was performed using the CFX96 Real-Time System (Bio-Rad, USA) using a two-step method: 95°C 2 min, followed by 40 cycles of 95°C 15 s, 60°C 1 min and then a melting curve was generated and analyzed. The tomato *CAC* gene was selected as internal standard for organ-specific expression [48], and the tomato *EF1α* gene was used as internal control under abiotic stress [49]. The comparative 2^-ΔΔCT^ method was used to analyze the relative gene expression levels [50]. Gene-specific primers used for quantitative RT-PCR are listed in S3 Table, and a standard curve by serial dilution was analyzed for each specific gene using WT tomato cDNA.

### Overexpression Vector Construction and Tomato Transformation

The coding region of *SlDEAD31* gene was amplified from WT tomato cDNA using the primers *Ov-SlDEAD31*-F/R (S3 Table), with PrimeSTAR HS DNA polymerase (TaKaRa, Dalian, China) under the following PCR procedure: 98°C for 5 min followed by 35 cycles of 30 s at 98°C, 15 s at 56°C and 3 min at 72°C, the PCR products were confirmed by DNA sequencing. Then *SlDEAD31* was cloned into the plant expression vector pBIN121 between the *Xba* I and *Sac* I sites, under the control of the cauliflower mosaic virus 35S promoter. Afterwards, the resultant construct pBIN121-*SlDEAD31* was transformed into tomato cv Ailsa Craig by *Agrobacterium tumefaciens* (strain LBA4404) by the freeze-thaw method [51]. Positive transgenic lines were screened for kanamycin (50 mg L^−1^) resistance. Genomic DNA of transgenic tomato plants was extracted according to the manufacturer's protocol of Genomic DNA Extraction Kit (Invitrogen, Shanghai, China), and was used for the detection of the presence of T-DNA by PCR with the primers *NPTII*-F/R (S3 Table). T2 seeds from the selected transgenic lines were germinated on a medium with kanamycin (50 mg L^−1^) and homozygous plants were selected for further study.

### Salinity and Drought Stress Tolerance Assays

Seeds of WT and homozygous T2 *SlDEAD31*-overexpressing tomato plants were surface-sterilized and sown on the MS medium. Then WT and transgenic tomato seeds of consistent germination were selected and transferred to MS medium containing 0 and 100 mM NaCl. Culture vessels containing the seeds were incubated in a growth chamber for 10 d. Then the seedlings were scanned and used for shoot and root growth measurements by ImageJ software. For high-salinity stress in soil, WT and transgenic seedlings were pre-grown under normal conditions for 15 d and transferred to a greenhouse. Then 8-week-old plants of uniform size were used for the salt tolerance assay. The plants were irrigated from the bottom of the pots with water containing 400 mm NaCl (200 mL) every 72 h for 21 d, followed by irrigating normal water for 5 d. Then the plants whose upper leaves were alive were considered to survive. Pictures were taken to record the phenotypes.

For the drought tolerance assay, 8-week-old WT and homozygous T2 transgenic tomato plants were used. The drought treatment consisted of withholding water for up to 21 d, then plants were rewatered for 5 d, and the plants that could recover were considered to survive. Pictures were taken to record the phenotypes.

### Evaluation of Stress Tolerance

The measurement of RWC was performed as in our previous report [43]. For chlorophyll (Chl) measurement, each leaf sample was randomly selected from three individual plants, then weighed, ground in liquid nitrogen, and extracted with 10 mL 80% aqueous acetone (v/v). The extract was centrifuged at 4000 g for 5 min and the absorbance of the supernatants at 663 and 646 nm was recorded using a lambda 900 scanning spectrophotometer (PerkinElmer). Total chlorophyll content was calculated using the formulas according to the method of Wellburn [52]: Total Chl (μg mL^-1^) = 20.29A_646_+8.02A_663_.

To measure the water loss rate under dehydration conditions, leaves of a similar size, age and position were detached from WT and transgenic plants, and placed on dry filter papers. The relative humidity was ~45% at room temperature during the dehydration period. The leaves were weighed every 20 min for 200 min.

The level of lipid peroxidation was measured in terms of malondialdehyde (MDA) content, a product of lipid peroxidation. Fresh tissue (0.2 g) was ground in liquid nitrogen and homogenised in 4 mL of 0.1% trichloroacetic acid (TCA). The homogenate was centrifuged at 15,000 g for 5 min. 4.0 mL of 20% TCA containing 0.5% thiobarbituric acid (TBA) was added to 1.0 mL aliquot of the supernatant. The mixture was incubated at 95°C in a hot water bath for 30 min and then the reaction was terminated in an ice bath. After centrifugation at 10,000 g for 10 min, the absorbance of the supernatant was recorded at 532 and 600 nm for the correction of non-specific turbidity. The MDA equivalent was calculated as follows [53]: MDA (nmol/mL FW) = ((A532-A600)/155, 000)×10^6^.

### Statistical Analysis

When appropriate, data were subjected to one-way analysis of variance (ANOVA) and differential expression data were regarded as statistically significant when passing the Dunnett’s test at P<0.05 level. In addition, we adopted an experiential threshold of twofold for analyzing stress induction or repression, and threefold for organ-specific expression. The expression levels were designated as ‘induced’, ‘repressed’ or ‘different’ only if such differences met the above criteria.

## Supporting Information

S1 Fig The nucleotide and deduced amino acid sequence of *SlDEAD30*. (DOCX)

S2 Fig The nucleotide and deduced amino acid sequence of *SlDEAD31*. (DOCX)

S3 Fig Expression profiles of *SlDEAD30* and *SlDEAD31* genes under circadian rhythm. (DOCX)

S4 Fig The relative expression levels of stress-related genes of WT and *SlDEAD31-*overexpressing plants under normal and salt stress conditions. (DOCX)

S1 Table Sequence similarities and molecular characterization of DEAD-box genes. (DOCX)

S2 Table Putative cis-elements enriched in the promoters of *SlDEAD30* and *SlDEAD31* genes. (DOCX)

S3 Table Specific primer sequences used for gene amplification, cloning procedure and quantitative RT-PCR analysis. (DOCX)
