# Supplementary material for: SlDEAD31, a Putative DEAD-Box RNA Helicase Gene, Regulates Salt and Drought Tolerance and Stress-Related Genes in Tomato
Source: PLoS One. 2015 Aug 4;10(8):e0133849. doi: 10.1371/journal.pone.0133849 (PMC4524616; doi:10.1371/journal.pone.0133849)
Supplement: S2 Fig — (DOCX) [file pone.0133849.s002.docx]

**
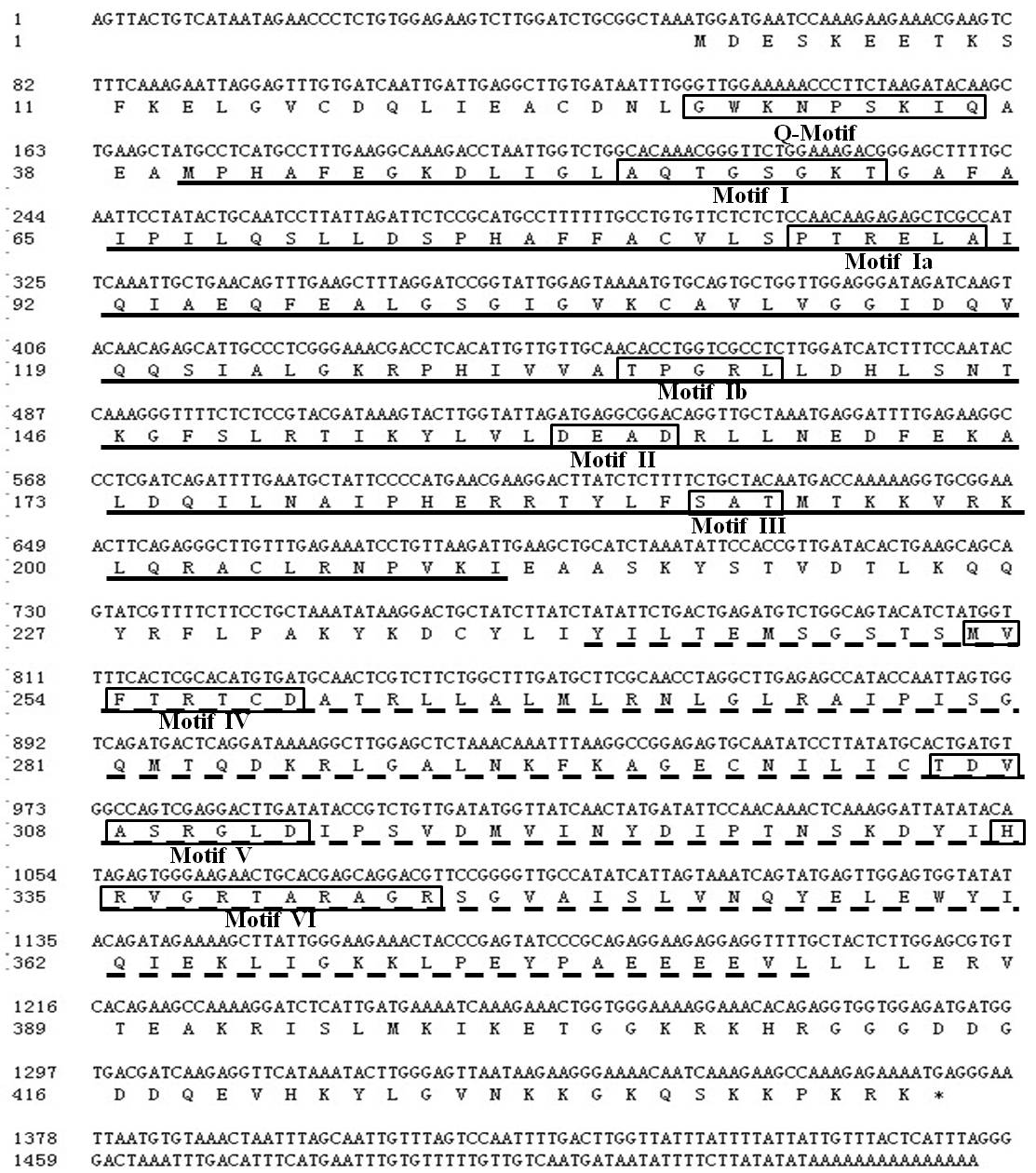
**

**S2 Fig. The nucleotide and deduced amino acid sequence of *SlDEAD31*.** All nine conserved helicase motifs (Q, I, Ia, Ib, II, III, IV, V, and VI) are shown in the boxes. The DEAD-box helicase superfamily domain is marked with a straight line and the helicase superfamily C-terminal domain (HELICc) with a dashed line.
