# Supplementary material for: SlDEAD31, a Putative DEAD-Box RNA Helicase Gene, Regulates Salt and Drought Tolerance and Stress-Related Genes in Tomato
Source: PLoS One. 2015 Aug 4;10(8):e0133849. doi: 10.1371/journal.pone.0133849 (PMC4524616; doi:10.1371/journal.pone.0133849)
Supplement: S3 Fig — (DOCX) [file pone.0133849.s003.docx]

**
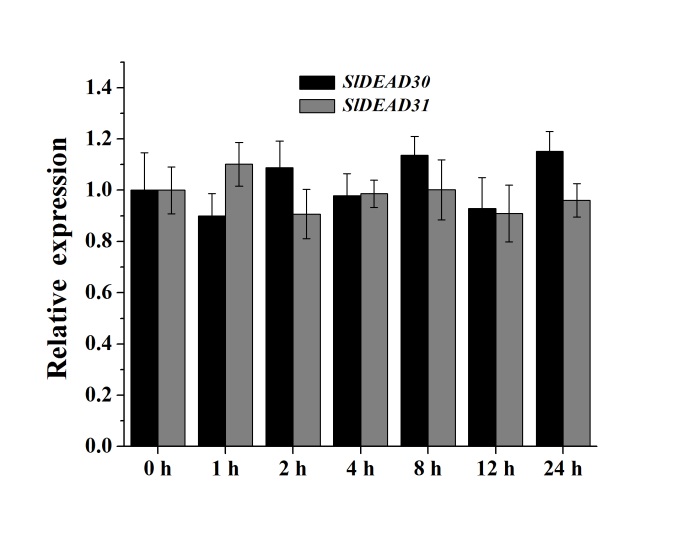
**

**S3 Fig. Expression profiles of *SlDEAD30* and *SlDEAD31* genes under circadian rhythm.** Gene expression was detected by RT-PCR using total RNA from leaves of 35-day-old tomato plants. Bars represent mean relative expression values ± SE (n=3). Values are presented relative to the plants at 0 h.
