# Supplementary material for: SlDEAD31, a Putative DEAD-Box RNA Helicase Gene, Regulates Salt and Drought Tolerance and Stress-Related Genes in Tomato
Source: PLoS One. 2015 Aug 4;10(8):e0133849. doi: 10.1371/journal.pone.0133849 (PMC4524616; doi:10.1371/journal.pone.0133849)
Supplement: S4 Fig — (DOCX) [file pone.0133849.s004.docx]

**
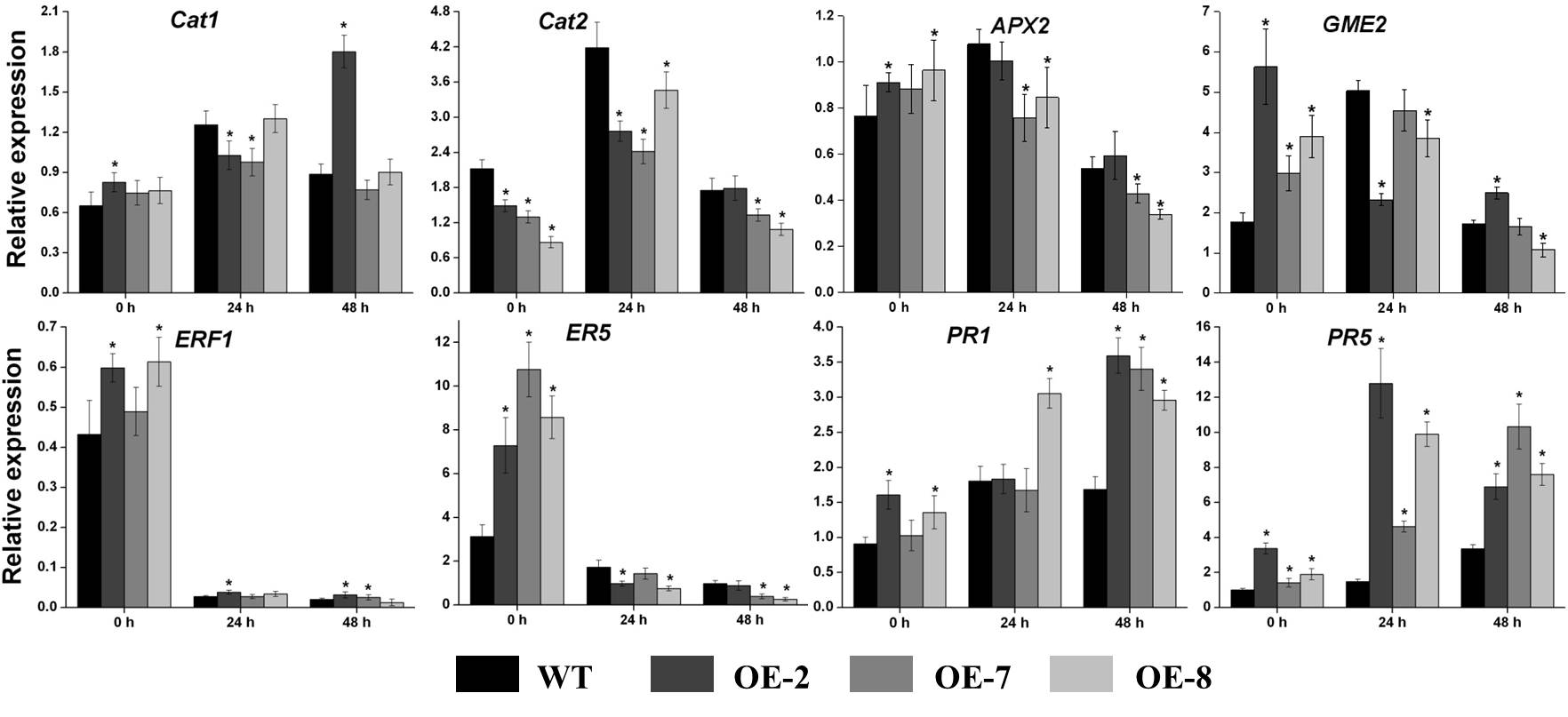
**

**S4 Fig. The relative expression levels of stress-related genes of WT and *SlDEAD31-*overexpressing plants under normal and salt stress conditions.** 8-week-old plants were treated with 400 mM NaCl, and leaf samples were harvested after 24 and 48 h. Seedlings harvested before salt stress were used as controls. Bars represent mean relative expression values ± SE (*n*=3). Asterisks indicate a significant difference (P<0.05) between WT and transgenic plants.
