# Supplementary material for: SlDEAD31, a Putative DEAD-Box RNA Helicase Gene, Regulates Salt and Drought Tolerance and Stress-Related Genes in Tomato
Source: PLoS One. 2015 Aug 4;10(8):e0133849. doi: 10.1371/journal.pone.0133849 (PMC4524616; doi:10.1371/journal.pone.0133849)
Supplement: S1 Table — (DOCX) [file pone.0133849.s005.docx]

**S1 Table**. **Sequence similarities and molecular characterization of DEAD-box genes**.

| Gene name | *SlDEAD30* | *SlDEAD31* | *AtRH9* | *AtRH36* | PI ^d^ | Mol wt. ( kDa) ^e^ | GRAVY ^f^ |
| --- | --- | --- | --- | --- | --- | --- | --- |
| *SlDEAD30* | **488** ^a^ | 42.9 ^b^ | 45.4 | 66.4 | 8.53 | 54.7 | -0.235 |
| *SlDEAD31* | *50.6* ^c^ | **439** | 79.9 | 42.6 | 9.16 | 48.9 | -0.294 |
| *AtRH9* | *49.5* | *71.5* | **456** | 42.9 | 9.07 | 51.1 | -0.374 |
| *AtRH36* | *67.4* | *51.3* | *49.6* | **491** | 9.00 | 54.8 | -0.298 |

^a^ Number of predicted amino acids (bold).

^b^ Sequence identities (%) of DEAD-box genes between amino acid sequences.

^c^ Sequence identities (%) of DEAD-box genes between nucleotide acid sequences (italic).

^d-f^ isoelectric point (pI), molecular weight (kDa), and grand average of hydropathicity (GRAVY) of the deduced polypeptide.
