# Supplementary material for: SlDEAD31, a Putative DEAD-Box RNA Helicase Gene, Regulates Salt and Drought Tolerance and Stress-Related Genes in Tomato
Source: PLoS One. 2015 Aug 4;10(8):e0133849. doi: 10.1371/journal.pone.0133849 (PMC4524616; doi:10.1371/journal.pone.0133849)
Supplement: S2 Table — (DOCX) [file pone.0133849.s006.docx]

**S2 Table**. **Putative cis-elements enriched in the promoters of *SlDEAD30* and *SlDEAD31* genes.**

| Genes | Cis-element name | Sequence | Site (s) |
| --- | --- | --- | --- |
|  | AGCBOXNPGLB | AGCCGCC | -125 |
|  | GCCCORE | GCCGCC | -124 |
|  | MYB2CONSENSUSAT | YAACKG | -58 |
|  | MYBCORE | CNGTTR | -28 |
|  | MYBCOREATCYCB1 | AACGG | -57 |
| *SlDEAD30* | MYCCONSENSUSAT | CANNTG | -720, -457, -302, -201 |
|  | WBBOXPCWRKY1 | TTTGACY | -199 |
|  | WBOXATNPR1 | TTGAC | -198 |
|  | WRKY71OS | TGAC | -755, -467, -453, -197 |
|  |  |  |  |
|  | MYB1AT | WAACCA | -575 |
|  | MYBATRD22 | CTAACCA | -576 |
|  | MYBCORE | CNGTTR | -78 |
|  | MYBST1 | GGATA | -550 |
| *SlDEAD31* | MYCATRD22 | CACATG | -435 |
|  | MYCCONSENSUSAT | CANNTG | -564, -451, -435, -142, -121 |
|  | WBBOXPCWRKY1 | TTTGACY | -383 |
|  | WBOXATNPR1 | TTGAC | -382 |
|  | WRKY71OS | TGAC | -381 |

W=A/T; Y=T/C; R=A/G; K=G/T; N=A/T/G/C
