# Supplementary material for: SlDEAD31, a Putative DEAD-Box RNA Helicase Gene, Regulates Salt and Drought Tolerance and Stress-Related Genes in Tomato
Source: PLoS One. 2015 Aug 4;10(8):e0133849. doi: 10.1371/journal.pone.0133849 (PMC4524616; doi:10.1371/journal.pone.0133849)
Supplement: S3 Table — (DOCX) [file pone.0133849.s007.docx]

**S3 Table**. **Specific primer sequences used for gene amplification, cloning procedure and quantitative RT-PCR analysis.**

| Primer code | | Accession number | | Primer sequences (5' →3') | Applications |
| --- | --- | --- | --- | --- | --- |
| *Ov-SlDEAD31*-F | | KJ713393 | | GCTCTAGAAGTTACTGTCATAATAGAACCCTC | To establish *SlDEAD31* overexpressing lines; added *Xba*I site underlined |
| *Ov-SlDEAD31*-R | |  | | CGAGCTCGACAACAAAAACACAAATTCAT | To establish *SlDEAD31* overexpressing lines; added *Sac*I site underlined |
| *F-SlDEAD30*-F | | KJ739798 | | TCTCCGATAACCGCAACTT | Full-length amplification for *SlDEAD30* |
| *F-SlDEAD30*-R | |  | | CCAAATCATATACATCTTAGCCAC |  |
| *NPTII*-F | |  | | GACAATCGGCTGCTCTGA | Positive transgenic plants detection |
| *NPTII*-R | |  | | AACTCCAGCATGAGATCC |  |
| *CAC*-Q-F | SGN-U314153 | | CCTCCGTTGTGATGTAACTGG | | Internal standard gene for Quantitative RT-PCR in tomato development |
| *CAC*-Q-R |  | | ATTGGTGGAAAGTAACATCATCG | |  |
| *EF1α*-Q-F | X53043 | | TACTGGTGGTTTTGAAGCTG | | Internal standard gene for Quantitative RT-PCR under abiotic stress |
| *EF1α*-Q-R |  | | AACTTCCTTCACGATTTCATCATA | |  |
| *SlNAC30-Q-F* | KJ739798 | | GCATTCATACAAGTCCCAGTCAC | | Quantitative RT-PCR analysis for *SlNAC30* |
| *SlNAC30-Q-R* |  | | ATGTCATAATTTACCACCAGATCAAC | |  |
| *SlNAC31-Q-F* | KJ713393 | | CATCTATGGTTTTCACTCGCAC | | Quantitative RT-PCR analysis for *SlNAC31*  Quantitative RT-PCR analysis for stress-related genes |
| *SlNAC31-Q-R* |  | | AGCCTTTTATCCTGAGTCATCTG | |  |
| *Cat1-Q-F* | M93719 | | AAATGGGTTGAGTCTTTATCCGA | |  |
| *Cat1-Q-R* |  | | TCATTGATTTTTCACATTGTAGGCT | |  |
| *Cat2-Q-F* | NM_001247257 | | TTCTGCCCTTCTATTGTGGTTC | |  |
| *Cat2-Q-R* |  | | GTGATGAGCACACTTTGGAGC | |  |
| *APX2-Q-F* | DQ099421 | | TCAGTGATCCTGCTTTCCGC | |  |
| *APX2-Q-R* |  | | TGTCACCACCCTCCCAACTCT | |  |
| *ERF1-Q-F* | AY192367 | | TTTTAGTATCGGATGGACG | |  |
| *ERF1-Q-R* |  | | GGCGGAGAAACAGAAGTA | |  |
| *GEM2-Q-F* | GQ150165 | | CCATCACATTCCAGGACCAGA | |  |
| *GEM2-Q-R* |  | | CGTAATCCTCAACCCATCCTTC | |  |
| *ER5-Q-F* | NM_001247009 | | TATTGGTAAAGATTGGGACATTGA | |  |
| *ER5-Q-R* |  | | TGTCTTCTTGTTTGTCACCGTTC | |  |
| *PR1-Q-F* | TC153553 | | TTGTCGAGGAAAATAAAATCCAG | |  |
| *PR1-Q-R* |  | | ACACACATCCAATAAAGCCCAC | |  |
| *PR5-Q-F* | AJ277064 | | AATGTTCTGTCCTTATGGCTCTACTC | |  |
| *PR5-Q-R* |  | | TGGCTACACTCATGATGAATCTACTTA | |  |
